# Renal and Cardiovascular Complications Following Type 2 Diabetes Mellitus in People With and Without HIV: Data From the Cohort Study on Morbidity and HIV in Sweden (COSMOHS) Between 2010 and 2024

**DOI:** 10.1093/cid/ciag261

**Published:** 2026-04-18

**Authors:** Isabela Killander Möller, Philippe Wagner, Johanna Brännström, Tintin Bäckdahl, Olof Elvstam, Magnus Gisslén, Pontus Hedberg, Åsa Mellgren, Fredrik Månsson, Pontus Nauclér, Josefin Nilsson, Piotr Nowak, Pär Sparén, Björn Eliasson, Christina Carlander

**Affiliations:** Department of Medicine Huddinge, Karolinska Institutet, Stockholm, Sweden; Department of Medical Epidemiology and Biostatistics, Karolinska Institutet, Stockholm, Sweden; Department of Clinical Sciences, Orthopedics, Lund University, Lund, Sweden; Centre for Clinical Research Västmanland, Västmanland County Hospital, Uppsala University, Västerås, Sweden; Department of Medicine Huddinge, Karolinska Institutet, Stockholm, Sweden; Department of Infectious Diseases, Södersjukhuset, Stockholm, Sweden; Department of Clinical Science and Education, Karolinska Institute, Södersjukhuset, Stockholm, Sweden; Department of Medicine Huddinge, Karolinska Institutet, Stockholm, Sweden; Department of Infectious Diseases, Karolinska University Hospital, Stockholm, Sweden; Department of Translational Medicine, Lund University, Malmö, Sweden; Department of Infectious Diseases, Växjö Hospital, Växjö, Sweden; Department of Infectious Diseases, Institute of Biomedicine, Sahlgrenska Academy at University of Gothenburg, Gothenburg, Sweden; Department of Infectious Diseases, Sahlgrenska University Hospital, Region Västra Götaland, Gothenburg, Sweden; Department of Medicine Huddinge, Karolinska Institutet, Stockholm, Sweden; Department of Infectious Diseases, Institute of Biomedicine, Sahlgrenska Academy at University of Gothenburg, Gothenburg, Sweden; Department of Infectious Diseases, Sahlgrenska University Hospital, Region Västra Götaland, Gothenburg, Sweden; Department of Translational Medicine, Lund University, Malmö, Sweden; Department of Infectious Diseases, Karolinska University Hospital, Stockholm, Sweden; Division of Infectious Diseases, Department of Medicine Solna, Karolinska Institutet, Stockholm, Sweden; Department of Medicine Huddinge, Karolinska Institutet, Stockholm, Sweden; Department of Medicine Huddinge, Karolinska Institutet, Stockholm, Sweden; Department of Infectious Diseases, Karolinska University Hospital, Stockholm, Sweden; Department of Medical Epidemiology and Biostatistics, Karolinska Institutet, Stockholm, Sweden; Department of Medicine, Sahlgrenska University Hospital, Region Västra Götaland, Gothenburg, Sweden; Department of Medicine Huddinge, Karolinska Institutet, Stockholm, Sweden; Department of Medical Epidemiology and Biostatistics, Karolinska Institutet, Stockholm, Sweden; Department of Infectious Diseases, Karolinska University Hospital, Stockholm, Sweden

**Keywords:** HIV, diabetes, diabetes complications, epidemiology

## Abstract

**Background:**

Type 2 diabetes mellitus (T2DM) is common among people with HIV (PWH), although studies comparing T2DM-related complications in people with and without HIV (PWoH) remain limited. This was assessed using data from the Cohort Study on Morbidity and HIV in Sweden (COSMOHS).

**Methods:**

Nationwide study including Swedish residents born 1930–2006, diagnosed with T2DM between 2010 and 2019, followed until 31 December 2024, using 7 national registers including national HIV and diabetes registers. Follow-up began at T2DM diagnosis. Outcomes included renal events, cardiovascular events, and all-cause mortality. Cox proportional regression estimated hazard ratios (adjHRs) by HIV status, stratified by propensity score quintiles of age, sex, migrant status, comorbidities, and socioeconomics.

**Results:**

350 PWH and 311 668 PWoH were included, with similar median follow-up time (PWH, 7.8 years; PWoH, 8.8 years). PWH had higher renal risk than PWoH (major adverse kidney event: adjHR, 2.04; 95% CI, 1.57-2.65) but no significantly increased cardiovascular risk (major adverse cardiovascular event: adjHR, 1.18; 95% CI, .87–1.60) or all-cause mortality. Findings were consistent across sensitivity analyses, including competing-risk models for death and excluding PWH receiving tenofovir disoproxil fumarate at baseline. Accounting for the benign plasma/serum-creatinine increase associated with bictegravir, cobicistat, dolutegravir, and rilpivirine, the increased renal risk remained, although not statistically significant. The renal risk was most prominent in PWH with BMI ≥30 kg/m^2^.

**Conclusions:**

In this nationwide cohort, PWH with T2DM exhibited higher risk of renal complications than PWoH, indicating enhanced renal monitoring may be warranted. Further research should investigate underlying mechanisms, including antiretroviral therapy, to guide clinical management.

## Teaser

Among people with type 2 diabetes mellitus (T2DM), people with HIV (PWH) experienced increased risk of renal complications compared with persons without HIV, while cardiovascular risk and mortality did not differ, highlighting the importance of intensified renal monitoring in PWH with T2DM.

## Graphical Abstract

**Graphical Abstract:**
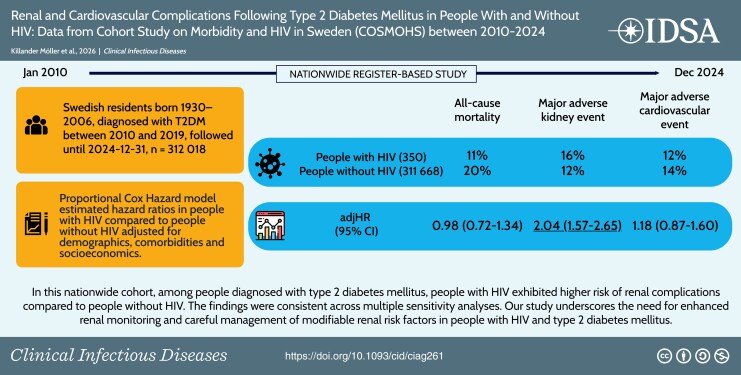
This graphical abstract is also available at Tidbit: https://tidbitapp.io/institutional-portal/clinical-infectious-diseases/tidbits/renal-and-cardiovascular-complications-following-type-2-diabetes-mellitus-in-people-with-and-without-hiv-data-from-cohort-study-on-morbidity-and-hiv-in-sweden-cosmohs-between-2010-2024/update

The incidence and prevalence of type 2 diabetes mellitus (T2DM) among people with human immunodeficiency virus (HIV; PWH) has been shown to be high, although varying globally [1], with estimated an prevalence of 9% in PWH [2] compared with 6% in the general population [3]. A recent US study found higher odds of converting from prediabetes to T2DM among men with HIV compared with men without HIV [4]. Central obesity [5], higher body mass index (BMI) [6], and non-White ethnicity [4] have shown to be associated with the incidence of T2DM in PWH. Additionally, integrase strand transfer inhibitors (INSTIs) have been suggested to be associated with weight gain [7] and increased risk of T2DM [6]. One study investigated the potential interaction between INSTI use and BMI and found no effect modification on the risk of T2DM, concluding that INSTI-associated weight gain had the same implications for T2DM risk as weight gain from other causes [6].

To date, it is yet unclear whether PWH face an increased risk of T2DM-related complications compared with persons without HIV (PWoH). Hypothetically, as PWH are followed regularly in specialist outpatient clinics in many countries, this may lead to earlier detection and treatment of T2DM, and consequently fewer T2DM-related complications. However, PWH tend to have lower socioeconomic status than PWoH [8], which is independently associated with a higher risk of developing T2DM, suboptimal glycemic control, and earlier onset of complications [9]. Therefore, while earlier T2DM diagnosis and access to healthcare potentially could favor PWH [8], these benefits may be offset by social vulnerability.

It has been suggested, based on glycated hemoglobin (HbA1c) measurements, that PWH have poorer glycemic control than PWoH following a recent T2DM diagnosis [10] and that PWH are younger at the time of T2DM diagnosis compared with PWoH [4]. Given that people with T2DM in the general population demonstrate lower life expectancy and increased all-cause mortality compared with those without T2DM, particularly when diagnosed with T2DM at younger ages [11], assessing T2DM-related complications in PWH is essential to inform targeted interventions and clinical guidelines.

By linking 7 Swedish nationwide registers, our aim was to study T2DM-related complications, including cardiovascular events, kidney events, and all-cause mortality following T2DM diagnosis in PWH compared with PWoH, while controlling for demographic characteristics, socioeconomic status, and comorbidities.

## METHODS

### Study Design and Population

**Figure 1.**
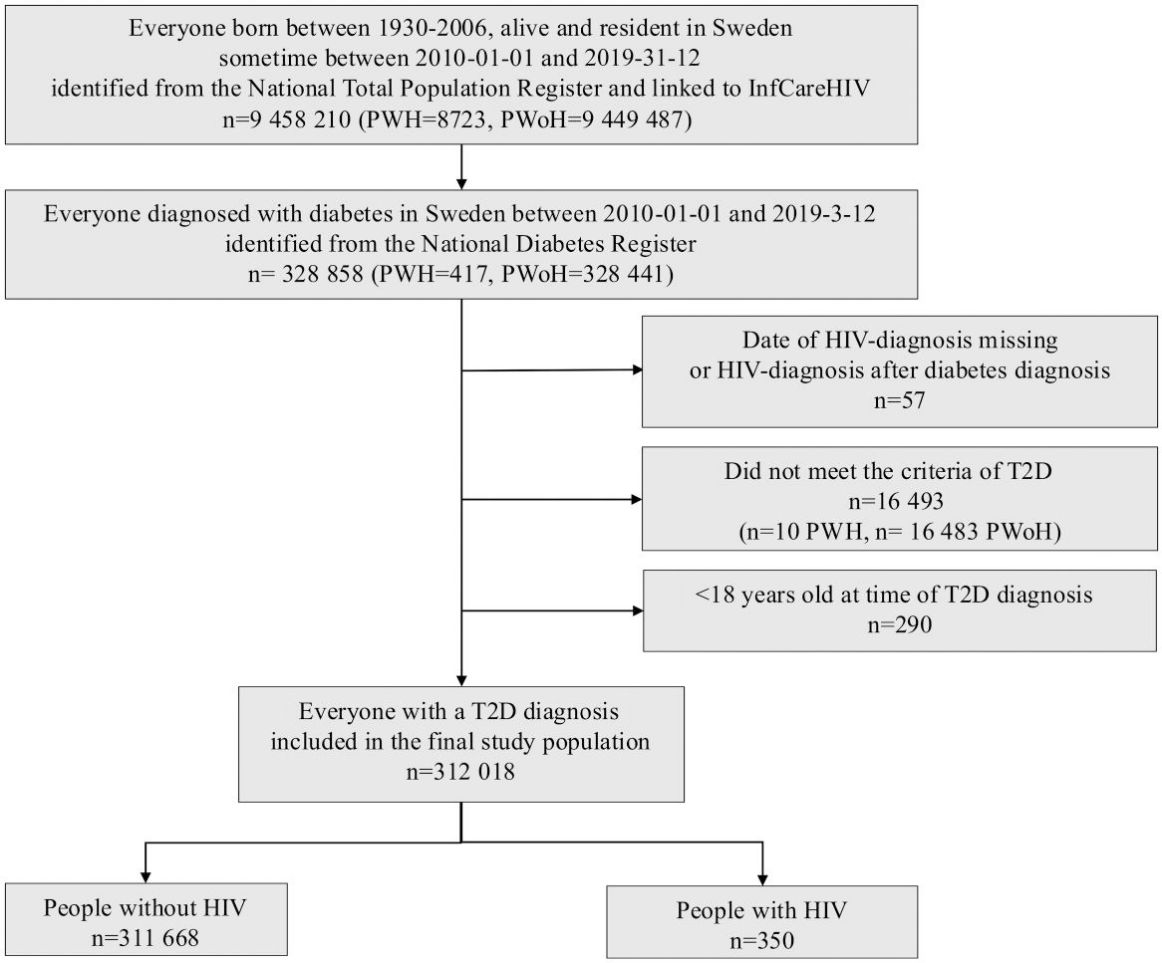
Flowchart of the study population. Abbreviations: HIV, human immunodeficiency virus; PWH, people with HIV; PWoH, people without HIV; T2DM, type 2 diabetes mellitus.

This was a nationwide, register-based study conducted as part of the Cohort Study on Morbidity and HIV in Sweden (COSMOHS). All individuals aged 18 years or older, born between 1930 and 2006, and diagnosed with T2DM between 1 January 2010 and 31 December 2019 were included. P with HIV diagnosed with HIV after T2DM diagnosis were excluded (Figure 1). The Swedish Total Population Register identified the study population through the Swedish personal identity number and linked to 7 nationwide registers described later [12]. Linkage was performed by the National Board of Health and Welfare, and pseudonymized files were delivered to the research group without the possibility of identifying individuals. People diagnosed with T2DM were identified from the National Diabetes Register, and date of diagnosis marked the start of follow-up. The end of follow-up was (1) outcome of interest (renal event, cardiovascular event, or mortality), (2) emigration, (3) death, or (4) end of study (31 December 2024), whichever came first. We used previously defined epidemiological criteria to define T2DM based on register data: (1) treatment with diet, with or without the use of oral antihyperglycemic agents, or (2) treatment with insulin, with or without the use of oral antihyperglycemic agents. The latter applied only to those aged 40 years and older at the time of T2DM diagnosis (ie, people <40 years only prescribed insulin were not classified as having T2DM to avoid misclassification of younger individuals with type 1 diabetes mellitus) [13].

### Data Sources/Materials

From the Swedish Total Population Register data on age, legal sex, country of birth, date of death, and migration status were retrieved [14], and from the Longitudinal Database for Integration and Labour Market Studies the level of education and income were collected [15]. Both of these registers are maintained by Statistics Sweden.

Three registers managed by the National Board of Health and Welfare were used. The National Patient Register contains diagnostic and procedure codes and dates for inpatient care since 1987 and specialist outpatient care since 2001 [16, 17]; all registered diagnoses of the study population were collected. Registered deaths and causes of death were obtained from the National Cause of Death Register, which contains data since 1961 [18]. Finally, we used the National Prescribed Drug Register, from which diabetic medications were collected [19].

Persons with HIV were identified from the Swedish National Quality Register for HIV (InfCareHIV), established in 2003 [20], which functions as a quality registry, a decision-support tool for physicians, and a research database. It includes data from 21 clinical centers in Sweden covering 99% of individuals diagnosed with HIV [21]. From InfCareHIV, HIV-related variables were retrieved, including date of HIV diagnosis, AIDS-defining diagnoses, CD4+ T-cell count, HIV RNA, mode of acquisition, and type of antiretroviral treatment (ART).

The National Diabetes Register is a quality registry established in 1999 encompassing data on persons with diabetes in Sweden. Approximately 85% of all individuals aged 18 years and older with diabetes are included [22]. From the National Diabetes Register, cases of T2DM, date of diagnosis, and laboratory measurements (such as HbA1c, BMI, plasma/serum-creatinine [P/S-creatinine], and estimated glomerular filtration rate [eGFR]) were retrieved.

### Outcomes

Primary outcomes were severe complications following T2DM diagnosis, including renal complications, cardiovascular complications, and all-cause mortality. Renal complications included acute kidney injury (AKI), a 40% or more decrease in eGFR from baseline, a 50% or more increase in P/S-creatinine from baseline, renal death, and major adverse kidney event (MAKE). Renal death was defined as 30-day mortality after either AKI, a 40% or more decrease in eGFR, or a 50% or more increase in P/S-creatinine; MAKE was the composite outcome of AKI, a 40% or more decrease in eGFR, or a 50% or more increase in P/S-creatinine. Cardiovascular complications included coronary heart disease (CHD), stroke, cardiovascular death, and major adverse cardiovascular event (MACE); MACE was the composite outcome of CHD, stroke, or cardiovascular death, as previously used [23].

Secondary outcomes included chronic kidney disease and heart failure. Acute kidney injury, CHD, and stroke were defined using *International Classification of Diseases, Tenth Revision* (ICD-10), codes from inpatient care, while chronic kidney disease and heart failure were defined using ICD-10 codes from both the inpatient care and outpatient care, as described in Supplementary Table 1.

### Other Study Variables

Variables included age, legal sex, migrant status (born outside Sweden), level of education, and income (age-specific income quartiles). Comorbidities were classified using ICD-10 codes registered 5 years prior to T2DM diagnosis (Supplementary Table 2). Comorbidity burden was classified as number of conditions, considering all items in the Charlson Comorbidity Index except for diabetes mellitus and AIDS [24].

Type 2 diabetes mellitus–related variables were the most recent value within 1 year after T2DM diagnosis. These included HbA1c (mmol/mol), BMI (kg/m^2^), blood pressure (diastolic and systolic, mmHg), eGFR (based on P/S-creatinine, mL/minute/1.73m^2^), P/S-creatinine (µmol/L), low-density lipoprotein (mmol/L), high-density lipoprotein (mmol/L), and triglycerides (mmol/L). The most common diabetic medication prescribed during the first year of initiation of diabetic medication was categorized and presented (Supplementary Table 3).

For PWH only, HIV-related variables are presented and described in detail in Supplementary Section 1. These included CD4+ T-cell count, nadir CD4+ T-cell count (the lowest ever measured before T2DM diagnosis), peak HIV RNA (copies/mL), viral suppression (HIV RNA <50 copies/mL within 2years of inclusion), mode of HIV acquisition, date of HIV diagnosis, antiretrovirals used at inclusion, and whether PWH switched to bictegravir (BIC), cobicistat (COB), dolutegravir (DTG), or rilpivirine (RPV) during the study period.

### Statistical Analysis

Baseline characteristics of PWH and PWoH were summarized and presented as medians (IQR), means (SD), or frequencies (percentages). Cox proportional hazard models estimated unadjusted hazard ratios (HRs) and adjusted HRs (adjHRs) and corresponding 95% CIs for the outcomes in PWH compared with PWoH. The model was stratified by quintiles of propensity score [25], calculated using logistic regression, which included age (categorized), sex, migrant (yes/no), specific comorbidities, number of comorbidities (0, 1, 2, 3, and ≥4), level of education, and income. This approach was adopted due to the limited number of outcomes among PWH. To determine which covariates to include in the model, a directed acyclic graph was created (DAGitty version 3.1) [26], although some uncertainty remains regarding the causal relationships between comorbidities, socioeconomic status, and HIV (Supplementary Figure 1).

Due to missing baseline values from the National Diabetes Register, BMI and HbA1c were not included in the main model, but 2 separate subanalyses excluding individuals with missing baseline BMI and HbA1c were performed. The propensity score was recalculated to include BMI (categorized: <25, 25–29.9, and ≥30 mg/m^2^), and adjustment was performed accordingly. A similar approach was applied for HbA1c (categorized: ≤47, 48–53, 54–58, 59–74, 75–85, ³86 mmol/mol).

### Sensitivity Analyses

First, we included first diabetic medication prescribed in the propensity score and adjusted for it accordingly. Second, we excluded PWH receiving tenofovir disoproxil fumarate (TDF) at time of T2DM diagnosis when assessing the renal outcomes, given the association between TDF and kidney toxicity [27]. Third, BIC, COB, DTG, and RPV cause an increase P/S-creatinine that remains stable over time, without affecting the GFR [28–31]. Therefore, we imputed adjusted P/S-creatinine values by subtracting 10 µmol/L for PWH who were not on BIC/COB/DTG/RPV at the time of T2DM diagnosis but initiated either drug during the study period. The outcome of a 50% or more increase in P/S-creatinine was then reassessed.

To account for the potential competing risk of death, cumulative incidences were presented of MACE, MAKE, and all-cause mortality. Further, we applied Fine-Gray subdistribution hazard models to estimate adjusted subdistribution HRs (adjSHRs) for MACE and MAKE.

Data management and analyses were performed in R version 4.5.1. The study was approved by the Swedish Ethical Review Authority (ref. nos 2023-00191-01 and 2024-04185-02).

## RESULTS

A total of 350 PWH and 311 668 PWoH were diagnosed with T2DM between 2010 and 2019 and included in the study (Figure 1). Persons with HIV were younger at diagnosis (median, 55 vs 63 years), more often migrants (60% vs 24%), and more frequently in the lowest income quartile (40% vs 25%), and a BMI of 30 kg/m^2^ or greater was less common in PWH (20% vs 32% (Table 1). The median time of follow-up was similar (PWH, 7.8 years; IQR, 6.0–10.3 years; PWoH, 8.8 years; IQR, 6.4–11.7 years).

**Table 1.** Baseline Characteristics at Time of Type 2 Diabetes Mellitus Diagnosis

	PWH (n = 350)	PWoH (n = 311 668)
Age, median [IQR], y	55 [47, 62]	63 [54, 71]
Age, categorized, n (%)		
≤39 y	15 (4.3)	11 068 (3.6)
40–49 y	104 (29.7)	37 621 (12.1)
50–59 y	124 (35.4)	70 567 (22.6)
60–69 y	67 (19.1)	97 772 (31.4)
≥70 y	40 (11.4)	94 640 (30.4)
Sex,^a^ n (%)		
Male	256 (73.1)	183 299 (58.8)
Migrant,^b^ n (%)		
Yes	208 (59.4)	76 167 (24.4)
Region of birth,^c^ n (%)		
Sweden	142 (40.6)	235 473 (75.6)
Asia and Pacific	22 (6.3)	11 393 (3.7)
Eastern and southern Africa	91 (26.0)	2628 (0.8)
Eastern Europe and Central Asia	3 (0.9)	9840 (3.2)
Latin America and the Caribbean	9 (2.6)	3169 (1.0)
Middle East and North Africa	16 (4.6)	18 530 (5.9)
Western/Central Europe and North America	28 (8.0)	29 664 (9.5)
Western and Central Africa	39 (11.1)	943 (0.3)
Unknown	0 (0.0)	28 (0.0)
Level of education,^d^ n (%)		
Primary (≤9 y)	104 (29.7)	91 728 (29.4)
Secondary (>9–12 y)	125 (35.7)	140 469 (45.1)
Tertiary (>12 y)	102 (29.1)	68 060 (21.8)
Missing	19 (5.4)	11 411 (3.7)
Level of income,^e^ n (%)		
Quartile 1	140 (40.0)	76 335 (24.5)
Quartile 2	86 (24.6)	76 265 (24.5)
Quartile 3	64 (18.3)	76 419 (24.5)
Quartile 4	49 (14.0)	76 772 (24.6)
Missing	11 (3.1)	5877 (1.9)
Comorbidities, n (%)		
Myocardial infarction	20 (5.7)	18 008 (5.8)
Congestive heart failure	7 (2.0)	11 801 (3.8)
Peripheral vascular disease	5 (1.4)	5166 (1.7)
Cerebrovascular disease	18 (5.1)	14 064 (4.5)
Chronic obstructive pulmonary disease	10 (2.9)	7545 (2.4)
Other chronic pulmonary disease	9 (2.6)	8026 (2.6)
Rheumatic disease	4 (1.1)	7992 (2.6)
Dementia	0 (0.0)	1304 (0.4)
Hemiplegia, tetraplegia	7 (2.0)	2027 (0.7)
Moderate or severe kidney disease	7 (2.0)	3675 (1.2)
Mild liver disease	7 (2.0)	1068 (0.3)
Moderate or severe liver disease	3 (0.9)	652 (0.2)
Viral hepatitis	31 (8.9)	1797 (0.6)
(Peptic) ulcer disease	2 (0.6)	2583 (0.8)
Any malignancy, including leukemia and lymphoma	20 (5.7)	19 168 (6.2)
Metastatic cancer	2 (0.6)	2501 (0.8)
Variables from NDR^f^		
HbA1c, median [IQR], mmol/mol	52.0 [44.5, 68.5]	50.0 [44.0, 62.0]
HbA1c (mmol/mol), categorized, n (%)		
≤47	88 (25.1)	85 738 (27.5)
48–53	42 (12.0)	49 059 (15.7)
54–58	23 (6.6)	20 093 (6.4)
59–74	40 (11.4)	28 642 (9.2)
75–85	17 (4.9)	10 647 (3.4)
≥86	29 (8.3)	23 175 (7.4)
Missing	111 (31.7)	94 314 (30.3)
BMI, mean (SD), kg/m^2^	28.2 (4.8)	31.2 (8.2)
BMI (kg/m^2^), categorized, n (%)		
<25	59 (16.9)	24 833 (8.0)
25–29.9	85 (24.3)	67 870 (21.8)
≥30	69 (19.7)	100 760 (32.3)
Missing	137 (39.1)	118 205 (37.9)
Blood pressure		
Systolic, mean (SD), mmHg	129.6 (16.1)	136.0 (16.8)
Diastolic, mean (SD), mmHg	80.5 (10.6)	80.4 (10.4)
Missing, n (%)	113 (32.3)	97 798 (31.4)
Albuminuria, categorized, n (%)		
Normal	139 (39.7)	127 652 (41.0)
Microalbuminuria	25 (7.1)	19 006 (6.1)
Macroalbuminuria	9 (2.6)	4196 (1.3)
Missing	177 (50.6)	160 814 (51.6)
P/S-creatinine, mean (SD), µmol/L	78.2 (34.8)	75.5 (24.9)
Missing, n (%)	132 (37.7)	106 070 (34.0)
Estimated glomerular filtration rate, mean (SD), mL/min/1.73 m^2^	92.6 (28.4)	87.4 (25.4)
Missing, n (%)	132 (37.7)	106 078 (34.0)
Triglycerides, mean (SD), mmol/L	2.7 (2.8)	2.1 (1.7)
Missing, n (%)	159 (45.4)	148 382 (47.6)
HDL, mean (SD), mmol/L	1.2 (0.3)	1.2 (0.4)
Missing, n (%)	151 (43.1)	136 095 (43.7)
LDL, mean (SD), mmol/L	3.0 (1.0)	3.1 (1.1)
Missing, n (%)	152 (43.4)	132 418 (42.5)
First prescribed diabetes medication,^g^ n (%)		
Acarbose	0 (0)	88 (<0.1)
DPP-4 inhibitors	9 (2.6)	8799 (2.8)
GLP-1 receptor agonists	9 (2.6)	4296 (1.4)
Meglitinides	4 (1.1)	1726 (0.6)
Metformin	241 (69)	233 796 (75)
SGLT2 inhibitors	10 (2.9)	6145 (2.0)
Sulfonylureas	10 (2.9)	6170 (2.0)
Thiazolidinediones	0 (0)	216 (<0.1)
Insulin	45 (13)	19 887 (6.4)
Missing/None	22 (6.3)	30 545 (9.8)

Percentages do not always add up to 100% due to rounding.

Abbreviations: BMI, body mass index; DPP-4, dipeptidyl peptidase-4; GLP-1, glucagon-like peptide 1; HbA1c, glycated hemoglobin; HDL, high-density lipoprotein; HIV, human immunodeficiency virus; IQR, interquartile range; LDL, low-density lipoprotein; NDR, National Diabetes Register; P/S-creatinine, plasma/serum creatinine; PWH, people with HIV; PWoH, people without HIV; SD, standard deviation; SGLT2 inhibitors, sodium-glucose cotransporter 2 inhibitors.

^a^Defined as legal sex, collected from the Total Population Register.

^b^Defined as born outside Sweden.

^c^Defined using UNAIDS categorization.

^d^Level of education was categorized as primary (<9 y), secondary (9–12 y), or tertiary (>12 y).

^e^Level of income categorized by age-specific annual income quartiles.

^f^Variables from the NDR retrieved within 1 y after diabetes diagnosis.

^g^Defined as the most common drug prescribed of the first year when diabetes medication was first prescribed.

Among PWH, 95% received ART, of whom 92% were virally suppressed (<50 copies/mL), and median CD4+ T-cell count was 583 cells/µL (IQR, 430–813) at T2DM diagnosis. A nadir CD4+ T-cell count of less than 200 cells/µL was observed in 52% and 17% had a previous AIDS-defining diagnosis. At T2DM diagnosis, 31% were treated with BIC or DTG, and during study, 19% and 51% later initiated BIC and DTG, respectively (Table 2).

**Table 2.** HIV-Related Characteristics of People With HIV Diagnosed With Type 2 Diabetes Mellitus

	People With HIV (n = 350)
Year of HIV diagnosis, n (%)	
1979–1999	164 (46.9)
2000–2009	118 (33.7)
2010–2014	56 (16.0)
2015–2020	12 (3.4)
Probable mode of HIV acquisition, n (%)	
MSM/bisexual	120 (34.3)
Heterosexual	192 (54.9)
IVDU	15 (4.3)
Mother to child	1 (0.3)
Blood products	6 (1.7)
Other or unknown	16 (4.6)
Current CD4+ T-cell count (cells/µL)^a^	
Continuous, median [IQR]	583 [430–813]
Categorized, n (%)	
<200	14 (4.0)
200–500	112 (32.0)
>500	206 (58.9)
Missing	18 (5.1)
Nadir CD4+ T-cell count (cells/µL)^b^	
Continuous, median [IQR]	190.0 [90.0, 281.0]
Categorized (<200), n (%)	182 (52.0)
HIV RNA (copies/mL)	
Peak HIV RNA ever, median [IQR]	133 500 [25 600–441 616]
Viral suppression^c^ (<50 copies/mL), n (%)	305 (91.9)
Previous AIDS diagnosis, n (%)	59 (16.9)
Antiretroviral treatment, n (%)	332 (94.9)
Nucleoside reverse transcriptase inhibitor backbone, n (%)	
Tenofovir disoproxil fumarate	116 (34.9)
Tenofovir alafenamide fumarate	55 (16.6)
Emtricitabine	166 (50.0)
Lamivudine	146 (44.0)
Abacavir	132 (39.8)
Didanosine	1 (0.3)
Zidovudine	6 (1.8)
Anchor drug, n (%)	
Integrase inhibitors	
Dolutegravir	101 (30.4)
Bictegravir	1 (0.3)
Elvitegravir	5 (1.5)
Raltegravir	37 (11.1)
Cabotegravir (long acting)	1 (0.3)
Boosted protease inhibitors^c^	
Darunavir	51 (15.4)
Atazanavir	29 (8.7)
Lopinavir	9 (2.7)
Nonnucleoside reverse transcriptase inhibitors	
Efavirenz	83 (25.0)
Nevirapine	13 (3.9)
Etravirine	20 (6.0)
Rilpivirine (oral)	23 (6.9)
Rilpivirine (long-acting)	1 (0.3)
Doravirine	83 (25.0)
Booster	
Cobicistat	14 (4.2)
Ritonavir	76 (22.9)
Entry inhibitors	
Maraviroc	2 (0.6)
Switched to bictegravir/cobicistat/dolutegravir/rilpivirine, n (%)	
Bictegravir	67 (19.1)
Cobicistat	21 (6.0)
Dolutegravir	179 (51.1)
Rilpivirine	24 (6.9)

Percentages do not always add up to 100% due to rounding. Data are at time of type 2 diabetes mellitus diagnosis unless otherwise specified.

Abbreviations: HIV, human immunodeficiency virus; IQR, interquartile range; IVDU, intravenous drug use; MSM, men who have sex with men.

^a^Current CD4+ T-cell count defined as the last CD4+ T-cells count one y prior to study start.

^b^Nadir CD4+ T-cell count, lowest CD4+ T-cell count measured since HIV was diagnosed.

^c^Of everyone receiving antiretroviral treatment.

**Figure 2.**
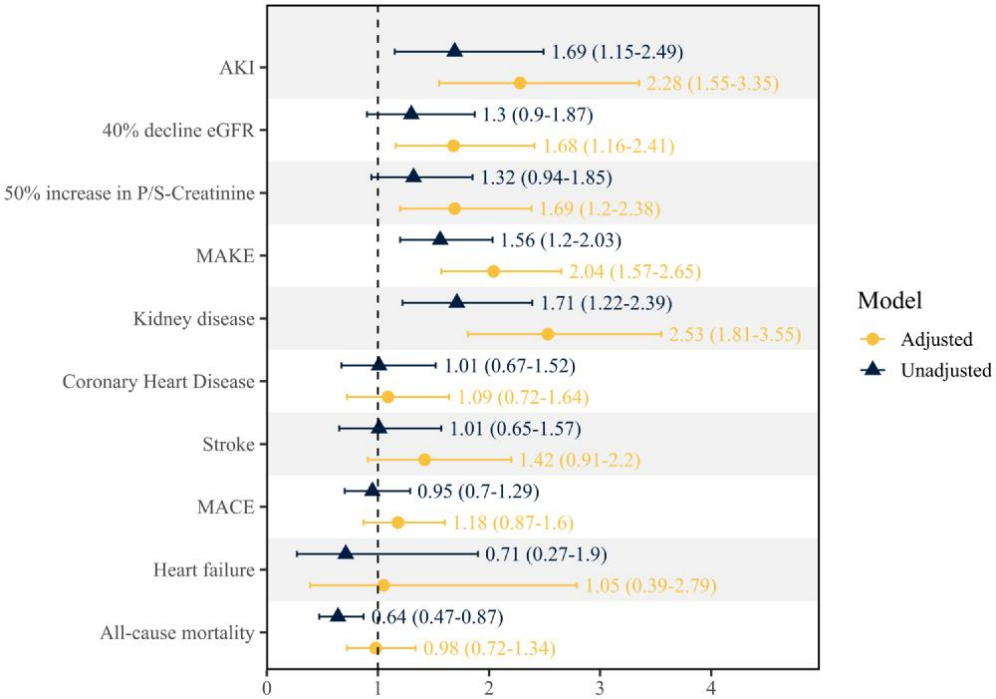
Hazard ratios and corresponding 95% CIs of outcomes after a T2DM diagnosis in PWH compared with PWoH. Abbreviations: AKI, acute kidney injury; CI, confidence interval; eGFR, estimated glomerular filtration rate; HIV, human immunodeficiency virus; MACE, major adverse cardiovascular event; MAKE, major adverse kidney event; P/S-creatinine, plasma/serum creatinine; PWH, people with HIV; PWoH, people without HIV; T2DM, type 2 diabetes mellitus.

All-cause mortality was 11% for PWH and 20% for PWoH (Table 3). However, there was no statistically significant difference between PWH and PWoH in the Cox proportional hazard model, stratified by quintiles of propensity score based on age, sex, migrant status, comorbidities, and socioeconomic status (adjHR, .98; 95% CI, .72–1.34) (Figure 2).

**Table 3.** Outcomes in People With HIV and Without HIV After Being Diagnosed With Type 2 Diabetes

	PWH (n = 350)	PWoH (n = 311 668)
Renal outcomes, n (%)		
Acute kidney injury	26 (7.4)	16 036 (5.1)
≥40% decline in eGFR	29 (8.3)	22 444 (7.2)
≥50% increase in P/S-creatinine	33 (9.4)	24 913 (8.0)
Renal death^a^	2 (0.6)	3181 (1.0)
Major adverse kidney event^b^	56 (16.0)	36 917 (11.8)
Kidney disease	34 (9.7)	20 518 (6.6)
Cardiovascular outcomes, n (%)		
Coronary heart disease	23 (6.6)	21 867 (7.0)
Stroke	20 (5.7)	19 285 (6.2)
Cardiovascular death^c^	4 (1.1)	7508 (2.4)
Major adverse cardiovascular event^d^	42 (12.0)	42 619 (13.7)
Heart failure	4 (1.1)	5772 (1.9)
Mortality, n (%)		
All-cause mortality	40 (11.4)	62 903 (20.2)

Percentages do not always add up to 100% due to rounding.

Abbreviations: eGFR, estimated glomerular filtration rate; HIV, human immunodeficiency virus; P/S-creatinine, plasma/serum creatinine; PWH, people with HIV; PWoH, people without HIV.

^a^Renal death was defined as 30-day mortality after acute kidney injury, ≥40% decline in eGFR, or ≥50% increase in P/S-creatinine.

^b^Major adverse kidney event was the composite outcome of acute kidney injury, 40% decline in eGFR, 50% increase in P/S-creatinine, or renal death.

^c^Cardiovascular death was defined as death due to either stroke or coronary heart disease.

^d^Major adverse cardiovascular event was the composite outcome of coronary heart disease, stroke or cardiovascular death.

Renal complications were more common in PWH than in PWoH (MAKE, 16% vs 12%) (Table 3). Persons with HIV had twice the risk of MAKE compared with PWoH (adjHR, 2.04; 95% CI, 1.57–2.65) (Figure 2), and the risk was statistically significantly increased for all renal complications. The cumulative incidence of MAKE was higher than that of all-cause mortality in PWH (Supplementary Figure 2). Accounting for the competing risk of death using the Fine-Gray subdistribution hazard model, MAKE remained statistically significantly increased in PWH compared with PWoH (adjSHR, 2.02; 95% CI, 1.55–2.63).

The proportion of PWH and PWoH with cardiovascular outcomes was similar, with only minor differences (MACE, 12% vs 14%) (Table 3). The adjHR was statistically nonsignificantly increased for PWH compared with PWoH (MACE: adjHR, 1.18; 95% CI, .87–1.60) (Figure 2). The cumulative incidence of MACE in PWH was lower compared with that of MACE and all-cause mortality in PWoH (Supplementary Figure 3). Applying the Fine-Gray subdistribution hazard model with all-cause mortality as a competing risk, the risk of MACE was nearly identical to the main analysis (adjSHR, 1.16; 95% CI, .86–1.56).

Analyzing only participants with available baseline BMI and including BMI in the model, PWH continued to show a higher risk of renal complications than PWoH, although estimates were attenuated (MAKE: adjHR, 1.81; 95% CI, 1.32–2.47) (Supplementary Figure 4). A comparable but statistically nonsignificant pattern was seen for cardiovascular complications. Findings were consistent in analyses restricted to participants with available HbA1c and including categorized HbA1c, with increased renal risk in PWH (MAKE: adjHR, 1.82; 95% CI, 1.35–2.45) but no statistically significant difference in cardiovascular risk (Supplementary Figure 5).

Hazard ratios were estimated within BMI categories (<25, 25–29.9, and ≥30 kg/m^2^) for all-cause mortality, MACE, and MAKE, and adjusted only for age due to few events to assess potential effect modification. All-cause mortality was similar by HIV status across the BMI categories (Supplementary Figure 6). The risks of MAKE were elevated in PWH compared with PWoH, showing an upward trend with increasing BMI category, with the highest hazard among those with a BMI of 30 kg/m^2^ or greater. However, PWH with a BMI less than 25 kg/m^2^ had a statistically significantly higher risk of MACE (adjHR, 2.37; 95% CI, 1.35–4.19) compared with PWoH in the same BMI category, while no significant associations were observed in the other BMI categories.

### Sensitivity Analyses

Additional analysis including diabetic medication in the model yielded similar results (Supplementary Figure 7). Excluding PWH receiving TDF at the time of T2DM diagnosis, PWH continued to demonstrate a statistically significantly higher risk of renal complications (MAKE: adjHR, 2.32; 95% CI, 1.70–3.18) (Supplementary Figure 8). The risk of a 50% or greater increase in P/S-creatinine was increased among PWH compared with PWoH in the main analysis (adjHR, 1.69; 95% CI, 1.20–2.38) (Figure 2). Imputing a 10-µmol/L lower P/S-creatinine value for PWH who were not on BIC/COB/DTG/RPV at the time of T2DM diagnosis but who initiated either drug during the study period, the adjHR for a 50% or greater increase in P/S-creatinine decreased (adjHR, 1.32; 95% CI, .90–1.94).

## DISCUSSION

This nationwide study, including all individuals diagnosed with T2DM in Sweden between 2010 and 2019, showed that PWH had a 2-fold increased risk of adverse kidney events compared with PWoH after adjustment for demographic, socioeconomic, and clinical factors through propensity score stratification. Furthermore, we observed no difference in all-cause mortality and a statistically nonsignificant higher cardiovascular risk in PWH with T2DM. These findings were largely consistent across multiple sensitivity analyses, including analyses restricted to individuals with complete BMI and HbA1c data, when excluding PWH receiving TDF at baseline, and incorporating competing risk models of death. After accounting for the benign P/S-creatinine increase associated with BIC, COB, DTG, and RPV, the renal risk was attenuated.

Given the benign increase in P/S-creatinine associated with BIC/COB/DTG/RPV, we assessed whether switching to any of these drugs during the study period influenced the results. The risk of a 50% or greater increase in P/S-creatinine remained elevated among PWH who switched, although the point estimate was lower and not statistically significant. This may suggest that, while ART-related creatinine elevation contributes to the association, it does not fully explain the observed higher risk of renal complications among PWH. Similarly, excluding individuals on TDF at T2DM diagnosis did not reduce the renal risk and, if anything, yielded slightly higher estimates.

The elevated risk of renal outcomes in PWH is concerning and may reflect a combination of factors. One study has demonstrated that PWH experienced poorer glycemic trajectories following T2DM diagnosis [10], which may contribute to an increased vulnerability of T2DM-related complications. However, additional adjustment for baseline HbA1c did not materially change the renal association, suggesting that baseline glycemic control alone is unlikely to explain the observed excess renal risk. Furthermore, baseline differences in kidney vulnerability between PWH and PWoH may also play a role as kidney disease is more prevalent in PWH, independent of diabetes, especially in people of African origin [32, 33]. However, it has been shown that individuals with both HIV and diabetes experience the greatest risk of chronic kidney disease compared with those with only 1 or neither condition [34]. Taken together, these findings underscore the need for intensified renal monitoring in PWH following a T2DM diagnosis.

Contrary to earlier findings, we did not identify a statistically significant increase in cardiovascular events among PWH compared with PWoH [35]. Importantly, nephropathy and cardiovascular disease represent different categories of T2DM complications, microvascular and macrovascular, respectively, and microvascular disease tends to occur earlier following a T2DM diagnosis [36]. This may explain why renal outcomes, but not cardiovascular outcomes, were elevated within our follow-up period. A larger sample of PWH in combination with longer follow-up may be required to detect cardiovascular differences. Furthermore, a recent study reported no increased risk of cardiovascular disease among PWH compared with PWoH when diabetes was controlled, and the authors suggested that HIV-specific factors may be less central to cardiovascular risk in high-risk groups such as people with T2DM, which may also explain our findings [37].

After controlling for BMI, the association between HIV status and renal events persisted. One study showed that weight gain confers a higher risk of T2DM in PWH compared with PWoH, raising the possibility that BMI modifies the relationship between HIV and T2DM-related complications [38]. In BMI-stratified analyses (<25, 25–29.9, and ≥30 kg/m^2^), PWH with a BMI less than 25 kg/m^2^ had a higher cardiovascular risk, whereas the renal risk increased progressively across BMI categories. This potentially reflects an effect modification; however, residual confounding cannot be excluded since the analyses were only adjusted for age.

Some limitations should be considered. Identification of T2DM through the National Diabetes Register (∼85% coverage) [22] may have resulted in more complete capture of PWH, given their regular specialist follow-up. Conversely, incomplete coverage among PWoH may have preferentially captured more severe diabetes cases. Together, these factors could bias complication risk estimates in both groups, potentially leading to either over- or underestimation. Baseline data from the National Diabetes Register, including BMI and P/S-creatinine, were missing for approximately one-third of the participants, which restricted complete adjustment for these covariates. As the number of PWH was relatively small, a detailed exploration of the association of different ART regimens was impeded. We did not have access to the underlying etiology of kidney injury, such as diabetic nephropathy or HIV-associated nephropathy, restricting conclusions of causal relations. Further, approximately 50% of PWH included were diagnosed before 1999, thus more likely to have been exposed to more nephrotoxic ART, potentially biasing our results. Our study lacked data on smoking, which may have acted as a confounder given that smoking tends to be more prevalent in PWH and is a risk factor for kidney disease. Finally, as this was a register-based study, a Swedish personal identity number was required, meaning that asylum seekers could not be included.

This study comes with several strengths, including nationwide coverage, long follow-up, and linkage across 7 high-quality registers, enabling comprehensive assessment of T2DM-related complications while considering demographic characteristics, socioeconomic status, and comorbidities. The diverse group of PWH, including a substantial proportion born outside Sweden and approximately one-third of whom were women, enhances the external validity. We identified an increased risk of renal complications among PWH compared with PWoH following T2DM diagnosis, and this association remained consistent across several sensitivity analyses, reinforcing the robustness of our findings and providing important insights for the clinical management of T2DM among PWH. Current European guidelines recommend annual screening with proteinuria [39], but it remains unclear how well these recommendations are followed in clinical practice and whether intensified renal monitoring alone is sufficient to prevent adverse renal events in PWH with T2DM. As noted by the American Diabetes Association, the only primary preventive strategies for chronic kidney disease in people with diabetes are glycemic control and blood pressure management [40]. Future studies in PWH with T2DM should therefore examine long-term trajectories of HbA1c, blood pressure, and albuminuria, as well as responses to therapeutic interventions.

### Conclusions

In conclusion, using nationwide register data spanning 2010–2024, we found that PWH experienced a markedly higher risk of renal complications after T2DM diagnosis compared with PWoH, whereas cardiovascular risk was elevated but not statistically significant, and all-cause mortality was similar between groups. These long-term data suggest that PWH may have increased susceptibility to kidney injury and future studies should disentangle the impact of specific ART regimens and examine longitudinal changes in metabolic and renal markers, such as blood pressure, BMI, albuminuria, and HbA1c. Meanwhile, our data suggest a need for intensified renal monitoring and careful management of modifiable renal risk factors in PWH with T2DM.

## Supplementary Material

ciag261_Supplementary_Data

## Supplementary Data

Supplementary materials are available at *Clinical Infectious Diseases* online. Consisting of data provided by the authors to benefit the reader, the posted materials are not copyedited and are the sole responsibility of the authors, so questions or comments should be addressed to the corresponding author.
