# Supplementary material for: Renal and Cardiovascular Complications Following Type 2 Diabetes Mellitus in People With and Without HIV: Data From the Cohort Study on Morbidity and HIV in Sweden (COSMOHS) Between 2010 and 2024
Source: Clin Infect Dis. 2026 Apr 18;82(6):e1252–61. doi: 10.1093/cid/ciag261 (PMC13341244; doi:10.1093/cid/ciag261)
Supplement: ciag261_Supplementary_Data [file ciag261_supplementary_data.pdf]

Renal and Cardiovascular Complications Following Type 2 Diabetes Mellitus in People With and Without HIV: Data from Cohort Study on Morbidity and HIV in Sweden (COSMOHS) between 2010-2024

Supplementary Material - CID-129977

.... 1
Supplementary Section 1. Detailed description of covariates. 3
Supplementary Table 1. Definitions of outcomes 4
Supplementary Table 2. Classification of comorbidities by ICD-10 codes 7
Supplementary Table 3. Diabetic medications by Anatomical Therapeutic Chemical Classification 12
Supplementary Figure 1. Directed Acyclic Graph of the exposure (HIV), outcome (T2DM-complications), and covariates. 13
Supplementary Figure 2. Cumulative incidence of MAKE and all-cause mortality in people with and without HIV. 14
Supplementary Figure 3. Cumulative incidence of MACE and all-cause mortality in people with and without HIV. 15
Supplementary Figure 4. Hazard ratio and corresponding 95% CIs of outcomes after a T2DM diagnosis in PWH compared to PWoH, including only people with a BMI at baseline. 16
Supplementary Figure 5. Hazard ratio and corresponding 95% CIs of outcomes after a T2DM diagnosis in PWH compared to PWoH, including only people with available HbA1c at baseline. 17
Supplementary Figure 6. Hazard ratio and corresponding 95% CIs of all-cause mortality, MACE, and MAKE after a T2DM diagnosis in PWH compared to PWoH categorized by BMI at baseline 18
Supplementary Figure 7. Hazard ratio and corresponding 95% CIs of outcomes after a T2DM diagnosis in PWH compared to PWoH with additional adjustment of diabetic medications. . 19
Supplementary Figure 8. Hazard ratio and corresponding 95% CIs of renal outcomes after a T2DM diagnosis in PWH compared to PWoH, excluding PWH on TDF at time of T2DM diagnosis. 20

Supplementary Section 1. Detailed description of covariates.

Age was categorized as 18-39, 40-49, 50-59, 60-69, and ≥70 years. Sex was defined as legal sex, typically assigned at birth but legal sex can be changed through a legal process, and our data reflects the legal sex registered at the time of data extraction. Migrant was defined as born outside Sweden. Education was categorized as primary (<9 years), secondary (9-12years), or tertiary (>12 years).

HIV-related variables

Current CD4+ T-cell count was the most recent value measured within two years of T2DM diagnosis and presented as both continuous and categorized (<200, 200-500, >500cells/µL). Nadir CD4+ T-cell count, i.e., the lowest registered CD4+ T-cell count before diabetes diagnosis, was presented as continuous and categorized (<200 or ≥200cells/µL). Peak HIV-RNA was defined as the highest registered viral load ever measured (copies/mL) before diabetes diagnosis. Viral suppression was defined as HIV-RNA <50 copies/mL within two years of inclusion. Mode of HIV acquisition was categorized as heterosexual, men who have sex with men or bisexual (MSM/bisexual), intravenous drug use (IVDU), mother to child, blood products, and unknown. Date of HIV diagnosis was categorized (1979-1999, 2000-2009, 2010-2015 and 2015-2020).

Supplementary Table 1. Definitions of outcomes

Outcome	ICD-10 codes	Definitions	Data source
Acute kidney injury (AKI)	N17.0, N17.1, N17.2, N17.8, N17.9, DR015 and DR023 (only if without chronic N18 kidney failure or N19.9)	Acute kidney failure, acute hemodialysis, or acute peritoneal dialysis.	The National Patient Register. Only inpatient care.
≥40% decline in eGFR		Compared to baseline. Based on P/S-creatinine.	The National Diabetes Register
≥50% increase in S-creatinine		Compared to baseline.	The National Diabetes Register
Renal death		Any death occurring within 30 days of AKI or 40% decline in eGFR	The Total Population Register, the National Cause of Death Register
Major adverse kidney event (MAKE)		Composite outcome of AKI, 40% decline in eGFR, 50% increase in P/S-creatinine, and renal death	
Chronic kidney disease	N03.2-N03.7, N05.2-N05.7, N11, N18, N19, I12.0, I13.1, Z49, Z94.0, Z99.2, DR012, DR013, DR016, DR024, DR060, DR061	Chronic/unspecified glomerulonephritis – not minimal change or focal, chronic pyelonephritis, hypertensive kidney disease with kidney failure, chronic/unspecified	The National Patient Register. Both inpatient and outpatient care.

		kidney failure, hypertensive heart and kidney disease with kidney failure, kidney transplant, hemodialysis, or peritoneal dialysis. Only outcomes if not had these ICD-10 codes at baseline.	
Coronary heart disease	I20.0, I21, I22, I23, I24	Unstable angina, acute myocardial infarction, re-infarction, complications to myocardial infarction, or other acute ischemic heart diseases	The National Patient Register. Only inpatient care.
Heart failure	I11.0, I13.0, I13.2, I25.5, I50	Hypertensive heart disease with or without kidney disease, ischaemic cardiomyopathy, and congestive heart failure. Only outcomes if not had these ICD-10 codes at baseline.	The National Patient Register. Both inpatient and outpatient care.
Stroke	I61, I62, I63, I64	Intracerebral hemorrhage, other nontraumatic intracranial hemorrhage, cerebral infarction, or	The National Patient Register. Only inpatient care.

		stroke, not specified as hemorrhage or infarction.	
Cardiovascular death	See codes for stroke and coronary heart disease	Any death due to coronary heart disease or stroke.	The National Cause of Death Register
Major adverse cardiovascular event (MACE)		Composite outcome of coronary heart disease, stroke, or cardiovascular death	
All-cause mortality		All with a death date	The Total Population Register, the National Cause of Death Register

Supplementary Table 2. Classification of comorbidities by ICD-10 codes

Condition	ICD-10 code
**Myocardial infarction**	I21 Acute myocardial infarction<br>I25.2 Old myocardial infarction<br>I22 Re-infarction)
**Congestive heart failure**	I11.0 Hypertensive heart disease<br>I13.0 Hypertensive heart and kidney disease<br>I13.2 Hypertensive heart and kidney disease with both (congestive) heart failure and kidney failure<br>I25.5 Ischaemic cardiomyopathyh<br>I42.0 Dilated cardiomyopathy<br>I42.6 Alcoholic cardiomyopathy<br>I42.7Cardiomyopathy due to drugs and other external agents<br>I42.8 Other cardiomyopathies<br>I42.9 Cardiomyopathy, unspecified<br>I43 Cardiomyopathy in infectious and parasitic diseases classified elsewhere<br>I50 Congestive heart failure
**Peripheral vascular disease**	I70 Atherosclerosis<br>I71 Aortic aneurysm and dissection<br>I73.1 Thrombangiitis obliterans, Thromboangiitis obliterans<br>I73.8 Other specified peripheral vascular diseases<br>I73.9 Peripheral vascular disease, unspecified<br>I77.1 Stricture of artery)<br>I79.0 Aneurysm of aorta in diseases classified elsewhere<br>I79.2 Peripheral angiopathy in diseases classified elsewhere<br>K55 Vascular disorders of the intestine
**Cerebrovascular disease**	G45 Transient cerebral ischaemic and related syndromes<br>I60 Subarachnoid hemorrhage<br>I61 Intracerebral hemorrhage

	I62 Other nontraumatic intracranial hemorrhage<br>I63 Cerebral infarction<br>I64 Stroke, not specified as haemorrhage or infarction<br>I67 Other cerebrovascular diseases<br>I69 Late complications from cerebrovascular disease
**Chronic obstructive pulmonary disease**	J43 Emphysema<br>J44 Other chronic obstructive pulmonary disease
**Other chronic pulmonary disease**	J41 Chronic bronchitis without airway obstruction<br>J42 Unspecified chronic bronchitis<br>J45 Asthma<br>J46 Acute severe asthma<br>J47 Bronchiectasis<br>J60 Coalworker's pneumoconiosis<br>J61 Pneumoconiosis due to asbestos and other mineral fibres<br>J62 Pneumoconiosis due to dust containing silica<br>J63 Pneumoconiosis due to other inorganic dusts<br>J64 Unspecified pneumoconiosis<br>J65 Pneumoconiosis associated with tuberculosis<br>J66 Airway disease due to specific organic dust<br>J67 Hypersensitivity pneumonitis due to organic dust<br>J68 Respiratory conditions due to chemicals, gases, fumes and vapours<br>J69 Pneumonitis due to solid or fluid substance<br>J70 Respiratory conditions due to other external agents
**Rheumatic disease**	M05, M06, M12.3 Rheumatoid arthritis<br>M07.0–3 Psoriatic arthritis<br>M08 Juvenile idiopathic arthritis<br>M13 Poly arthritis<br>M30 Polyarteritis nodosa<br>M31.3-M31.6 Necrotizing vasculitides including giant cell<br>arteritis

	M32 SLE<br>M33 Dermatopolymyosit, Dermato- and polymyositis<br>M34 Systemic sclerosis<br>M35.0 Sjögren syndrome<br>M35.1 Other overlap syndromes<br>M35.3 Polymyalgia rheumatica<br>M45-46 Ankylosing spondylitis
**Dementia**	F00 Dementia in Alzheimer’s disease<br>F01 Vascular dementia<br>F02 Dementia in other diseases classified elsewhere<br>F03 Unspecified dementia<br>F05.1 Delirium superimposed on dementia<br>G30 Alzheimer’s disease<br>G31.1 Senile degeneration of brain, not elsewhere classified<br>G31.9 Degenerative disease of the nervous system
**Hemiplegia, tetraplegia**	G11.4 Hereditary spastic paraplegia<br>G80 Cerebral palsy<br>G81 Hemiplegia<br>G82 Paraplegia and Tetraplegia<br>G83.0-G83.3Monoplegia to tetraplegia<br>G83.8 Tetraplegia
**Moderate or severe kidney disease**	N03.2-N03.7 Chronic glomerulonephritis – not minimal change or focal<br>N05.2-N05.7 Unspecified glomerulonephritis – not minimal change or focal<br>N11 Chronic pyelonephritis<br>N18 Chronic kidney failure<br>N19 Unspecified kidney failure<br>N25.0 Renal osteodystrophy resulting from impaired renal tubular function<br>I12.0 Hypertensive kidney disease with kidney failure

	I13.1 Hypertensive heart and kidney disease with kidney failure<br>Q61.1-Q61.4 Autosomal polycystic kidney disease<br>Z49 Preparatory care for dialysis, Extracorporeal/Other dialysis<br>Z94.0 Kidney transplant<br>Z99.2 Need for kidney dialysis<br>DR012 Control CAPD (continuous ambulatory peritoneal dialysis<br>DR013 Start CAPD<br>DR016 Hemodialysis chronic<br>DR024 peritoneal dialysis chronic<br>DR060 Home hemodialysis control<br>DR061 Home hemodialysis control
**Mild liver disease**	K70.3 Alcoholic liver disease, unspecified<br>K73 Chronic hepatitis, not elsewhere classified<br>K74.6 Fibrosis and liver cirrhosis<br>K70.3 Alcoholic liver cirrhosis<br>K75.4 Autoimmune hepatits
**Moderate or severe liver disease**	R18 +Any code for mild liver disease in combination with ascites<br>I85.0 Oesophageal varices with bleeding<br>I85.9 Oesophageal varices without bleeding<br>I98.2 Oesophageal varices without bleeding, in diseases classified elsewhere<br>I98.3 Oesophageal varices with bleeding, diseases classified elsewhere
**Viral hepatitis**	B15-B19 Viral hepatitis
**(Peptic) ulcer disease**	K25 Gastric ulcer<br>K26 Duodenal ulcer<br>K27 Peptic ulcer, site unspecified<br>K28 Gastrojejunal ulcer

**Any malignancy, including leukemia and lymphoma**	C00-C97, Not C77-80 and C87
**Metastatic cancer**	C77 Secondary and unspecified malignant neoplasm of the lymph nodes<br>C78 Secondary malignant neoplasm of the respiratory and digestive organs<br>C79 Secondary malignant neoplasm of other sites<br>C80 Malignant neoplasm without specification of site

All codes retrieved from the National Patient Register, both the inpatient and outpatient care. Only consider diagnoses sometime between 5 years before inclusion and up to date of inclusion. ICD-10: International Classification of Diseases, tenth revision.

Supplementary Table 3. Diabetic medications by Anatomical Therapeutic Chemical Classification

Diabetic medication	ATC-codes
**Acarbose**	A10BF, A10BD17
**DPP-4 inhibitors**	A10BH<br>In combination with other agents: A10BD07-13, A10BD19, A10BD21, A10BD24, A10BD25, A10BD27-30,
**GLP-1 receptor agonists**	A10BJ
**Meglitinides**	A10BX02, A10BX03, A10BX08, A10BD14
**Metformin**	A10BA02<br>In combinations with other agents: A10BD02, A10BD03, A10BD05, A10BD07, A10BD08, A10BD10, A10BD11, A10BD13, A10BD14, A10BD15, A10BD16, A10BD17, A10BD18, A10BD20, A10BD22, A10BD23, A10BD25, A10BD26, A10BD27, A10BD28
**SGLT2 inhibitors**	A10BK<br>In combination with other agents: A10BD15, A10BD16, A10BD19, A10BD20, A10BD21, A10BD23, A10BD24, A10BD25, A10BD27, A10BD29, A10BD30
**Sulfonylureas**	A10BB, A10BD01, A10BD02, A10BD04, A10BD06, A10BD31
**Thiazolidinediones**	A10BG<br>In combination with other agents: A10BD03-6, A10BD09, A10BD12, A10BD26
**Insulin**	Short acting: A10AB<br>Intermediate acting: A10AC<br>Premixed A10AD<br>Insulin Long acting: A10AE

All codes retrieved from the National Precribed Drug Register.International Classification of Diseases, tenth revision.

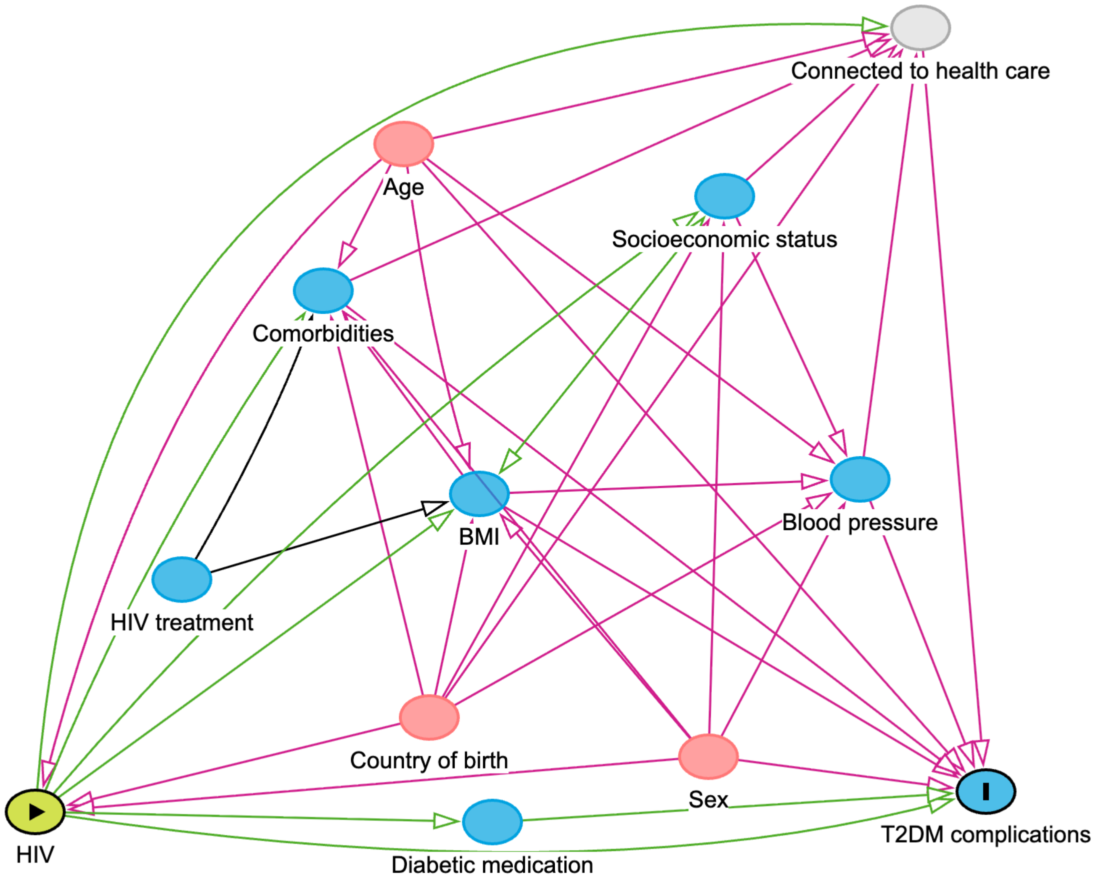

Supplementary Figure 1. Directed Acyclic Graph of the exposure (HIV), outcome (T2DM-complications), and covariates.

Abbreviations: BMI, body mass index; HIV, human immunodeficiency virus; T2DM, type 2 diabetes mellitus.

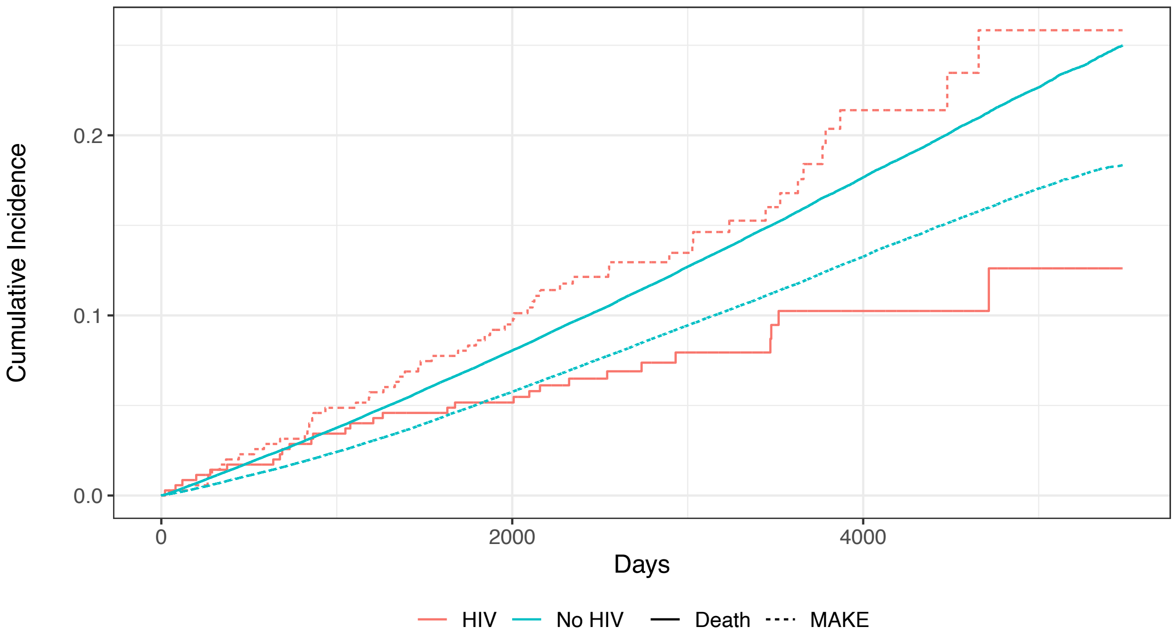

	0	2000	4000
**HIV**			
At Risk	350	274	62
Events	0	51	82
**No HIV**			
At Risk	311668	256403	87579
Events	8	42801	77882

Supplementary Figure 2. Cumulative incidence of MAKE and all-cause mortality in people with and without HIV.

MAKE defined as the composite outcome of acute kidney injury, ≥40% decrease of estimated glomerular filtration rate, or ≥50% increase of P/S-creatinine, or renal death.

Abbreviations: HIV, human immunodeficiency virus; MAKE, major adverse kidney event.

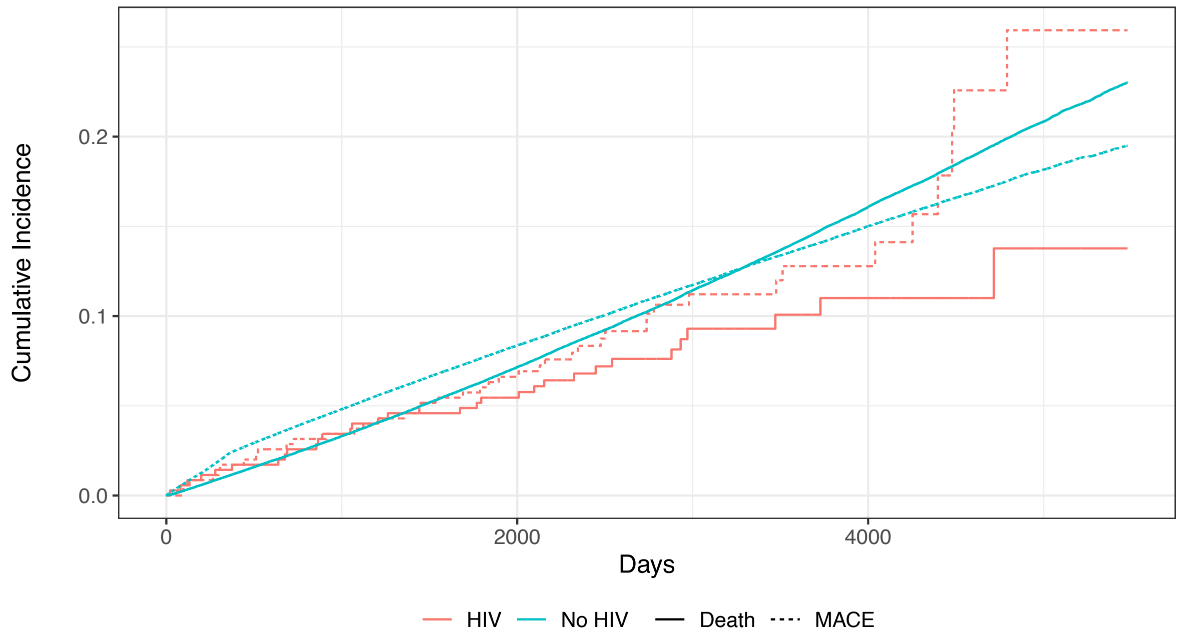

	0	2000	4000
**HIV**			
At Risk	350	281	67
Events	0	42	66
**No HIV**			
At Risk	311668	250832	85402
Events	8	48129	79835

Supplementary Figure 3. Cumulative incidence of MACE and all-cause mortality in people with and without HIV.

MACE defined as the composite outcome of coronary heart disease, stroke, or cardiovascular death.

Abbreviations: HIV, human immunodeficiency virus; MACE, major adverse cardiovascular event.

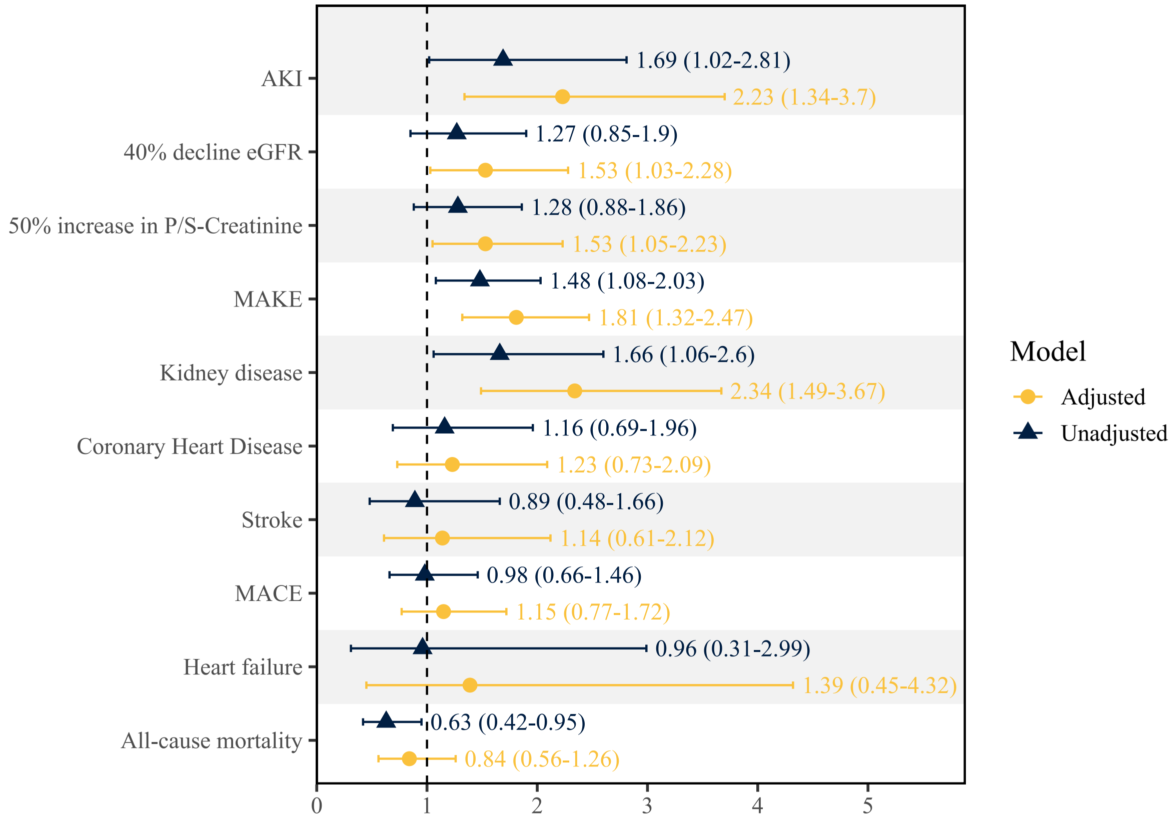

Supplementary Figure 4. Hazard ratio and corresponding 95% CIs of outcomes after a T2DM diagnosis in PWH compared to PWoH, including only people with a BMI at baseline.

Calculated using Cox proportional regression models and adjusted for quintiles of propensity score of age(categorized), sex, migrant, number of comorbidities, specific comorbidities, level of income, level of education, and categorized BMI (<25, 25-29,9, and ≥30kg/m^2).

MACE defined as the composite outcome of coronary heart disease, stroke, or cardiovascular death. MAKE defined as the composite outcome of acute kidney injury, ≥40% decline of eGFR, ≥50% increase of P/S-creatinine, or renal death.

Abbreviations: AKI, acute kidney injury; BMI, body mass index; CI, confidence interval; eGFR, estimated glomerular filtration rate; HR, hazard ratio; MACE, major adverse cardiovascular event; MAKE, major adverse kidney event; PWH, people with HIV; PWoH, people without HIV.

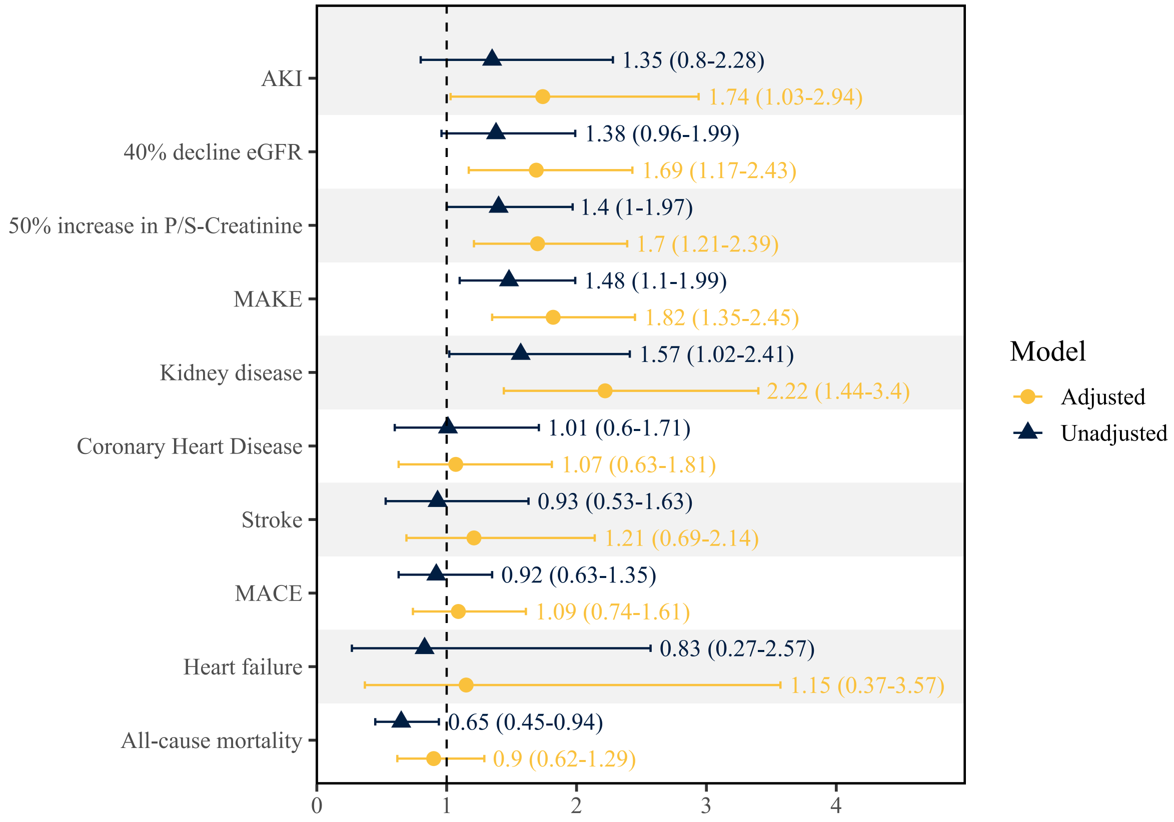

Supplementary Figure 5. Hazard ratio and corresponding 95% CIs of outcomes after a T2DM diagnosis in PWH compared to PWoH, including only people with available HbA1c at baseline.

Calculated using Cox proportional regression models and adjusted for quintiles of propensity score of age(categorized), sex, migrant, number of comorbidities, specific comorbidities, level of income, level of education, and categorized HbA1c (≤47, 48-53, 54-58, 59-74, 75-85, ≥86mmol/mol).

MACE defined as the composite outcome of coronary heart disease, stroke, or cardiovascular death. MAKE defined as the composite outcome of acute kidney injury, ≥40% decline of eGFR, ≥50% increase of P/S-creatinine, or renal death.

Abbreviations: AKI, acute kidney injury; CI, confidence interval; eGFR, estimated glomerular filtration rate; HR, hazard ratio; MACE, major adverse cardiovascular event; MAKE, major adverse kidney event; PWH, people with HIV; PWoH, people without HIV.

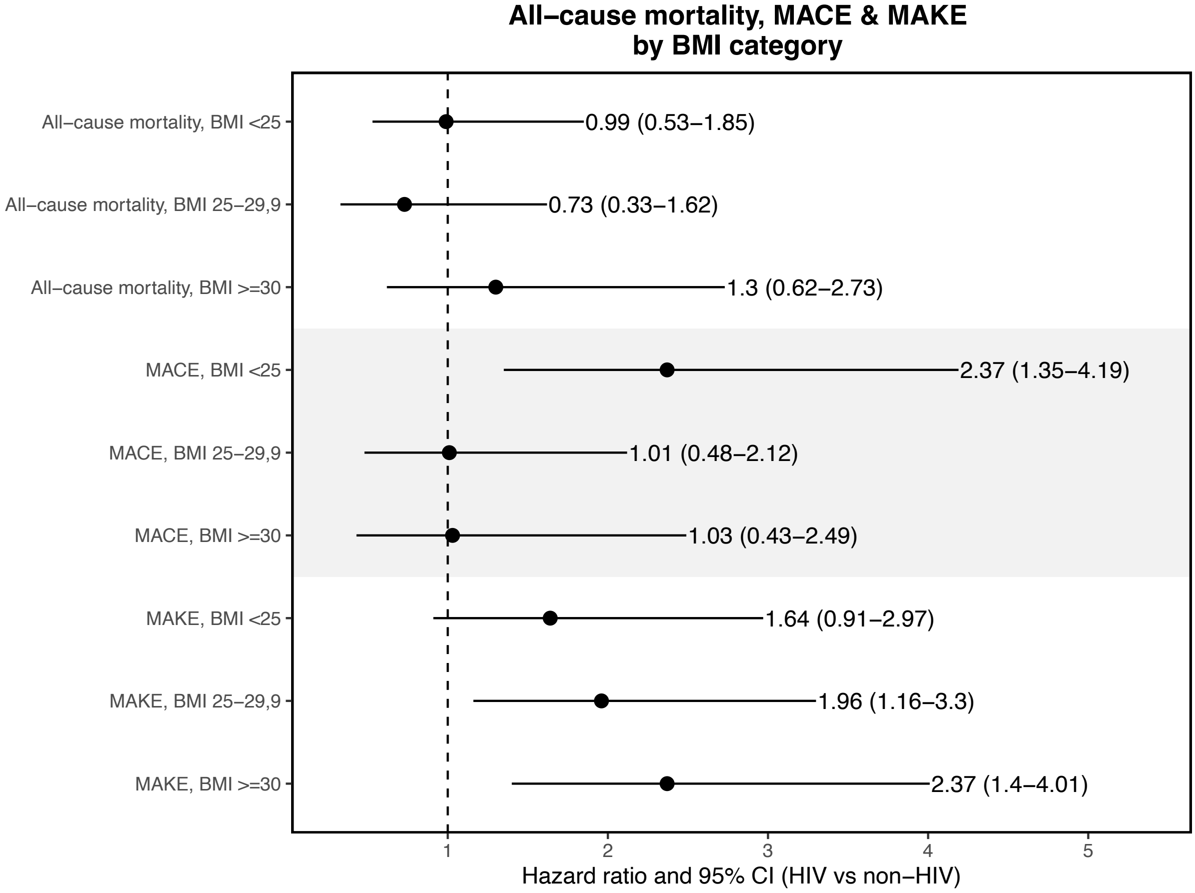

Supplementary Figure 6. Hazard ratio and corresponding 95% CIs of all-cause mortality, MACE, and MAKE after a T2DM diagnosis in PWH compared to PWoH categorized by BMI at baseline

Calculated using cox proportional regression models and adjusted for age(categorized). BMI categorized as <25, 25-29,9, and ≥30kg/m^2.

MACE defined as the composite outcome of coronary heart disease, stroke, or cardiovascular death. MAKE defined as the composite outcome of acute kidney injury, ≥40% decline of eGFR, ≥50% increase of P/S-creatinine, or renal death.

Abbreviations: BMI, body mass index; CI, confidence interval; eGFR, estimated glomerular filtration rate; HIV, human immunodeficiency virus; HR, hazard ratio; MACE, major adverse cardiovascular event; MAKE, major adverse kidney event; PWH, people with HIV; PWoH, people without HIV.

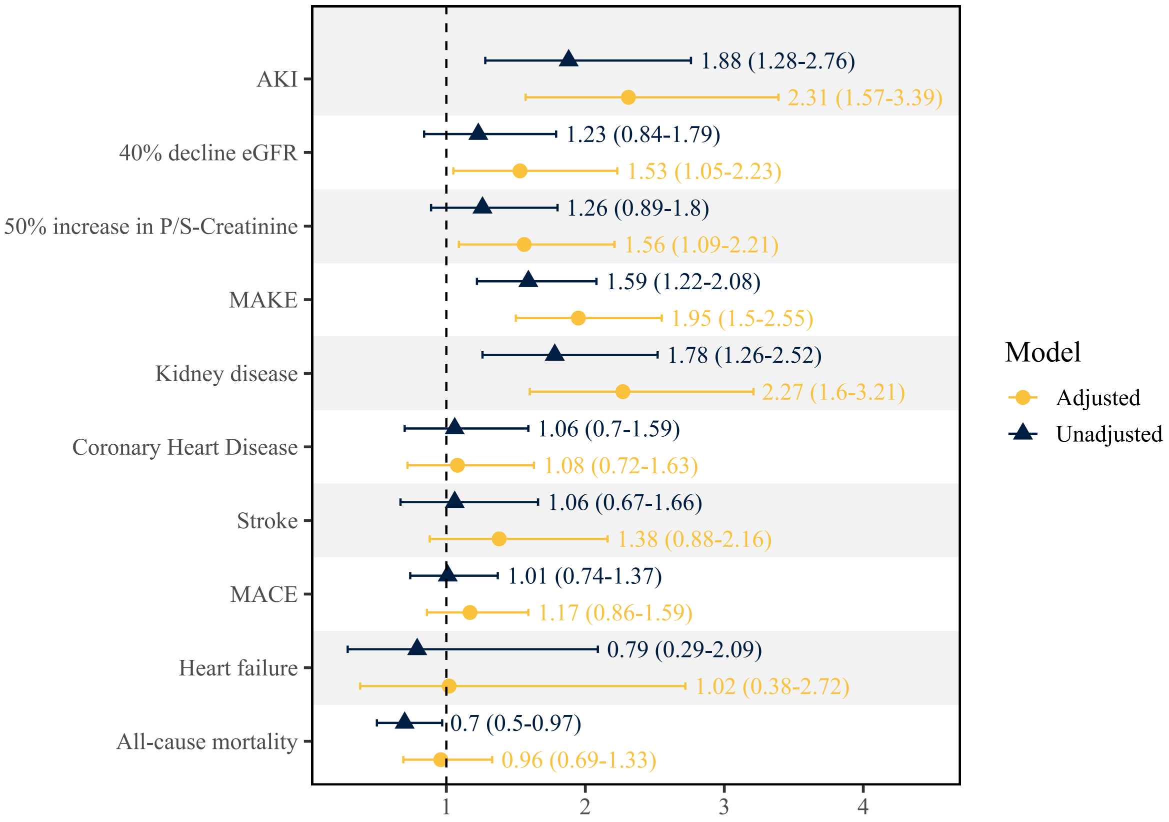

Supplementary Figure 7. Hazard ratio and corresponding 95% CIs of outcomes after a T2DM diagnosis in PWH compared to PWoH with additional adjustment of diabetic medications.

Calculated using Cox proportional regression models and adjusted for quintiles of propensity score of age (categorized), sex, migrant, number of comorbidities, specific comorbidities, level of income, level of education, and type of antidiabetic medication.

MACE defined as the composite outcome of coronary heart disease, stroke, or cardiovascular death. MAKE defined as the composite outcome of acute kidney injury, ≥40% decline of eGFR, ≥50% increase of P/S-creatinine, or renal death. Diabetic medication considered was the most common agent prescribed during the first year of initiation of diabetic medication.

Abbreviations: AKI, acute kidney injury; BMI, body mass index; CI, confidence interval; eGFR, estimated glomerular filtration rate; HR, hazard ratio; MACE, major adverse cardiovascular event; MAKE, major adverse kidney event; PWH, people with HIV; PWoH, people without HIV.

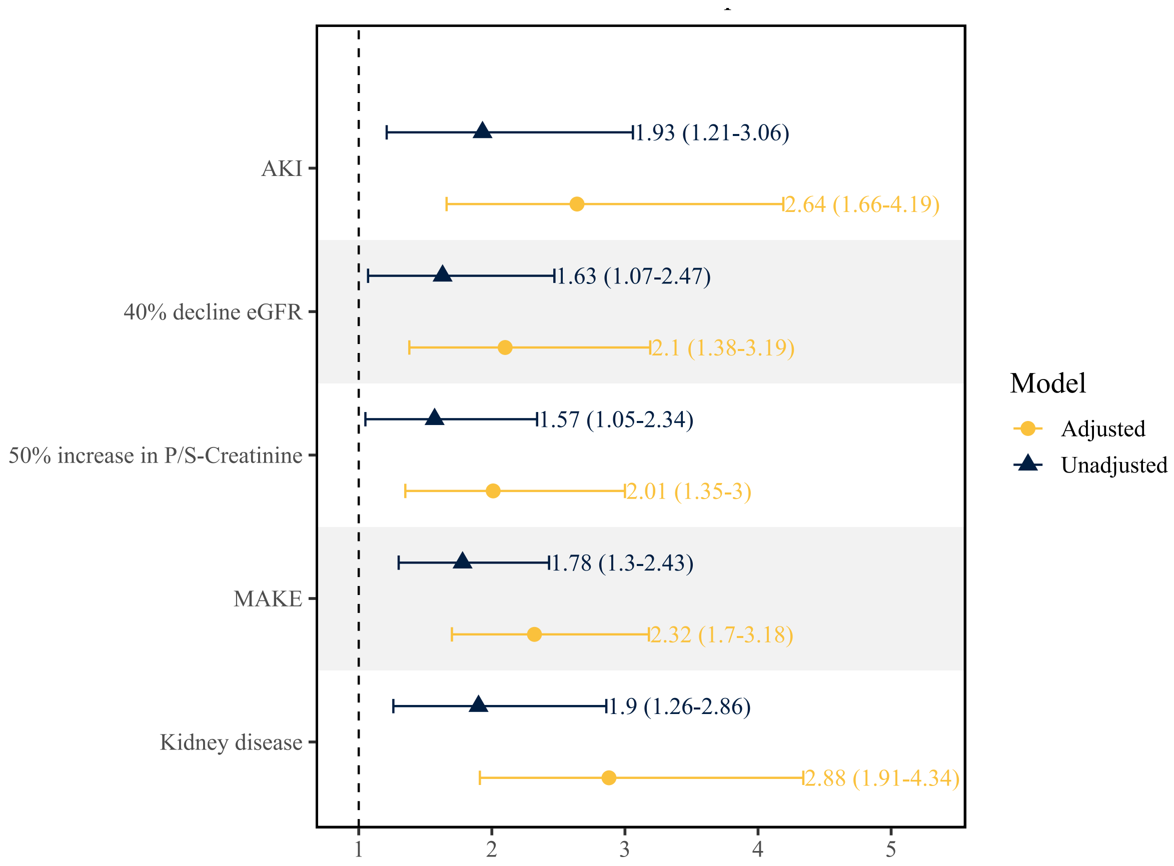

Supplementary Figure 8. Hazard ratio and corresponding 95% CIs of renal outcomes after a T2DM diagnosis in PWH compared to PWoH, excluding PWH on TDF at time of T2DM diagnosis.

Calculated using Cox proportional regression models and adjusted for quintiles of propensity score of age (categorized), sex, migrant, number of comorbidities, specific comorbidities, level of income, and level of education. MAKE defined as the composite outcome of acute kidney injury, ≥40% decline of eGFR, ≥50% increase of P/S-creatinine, or renal death.

Abbreviations: AKI, acute kidney injury; CI, confidence interval; eGFR, estimated glomerular filtration rate; HR, hazard ratio; MAKE, major adverse kidney event; PWH, people with HIV; PWoH, people without HIV.
